# Association between fat mass and obesity-related variant and osteoarthritis risk: Integrated meta-analysis with bioinformatics

**DOI:** 10.3389/fmed.2022.1024750

**Published:** 2022-09-23

**Authors:** Kun Zhao, Liuyan Nie, Grace Min Jun Chin, Xiangming Ye, Peng Sun

**Affiliations:** ^1^Department of Rehabilitation Medicine, Center for Rehabilitation Medicine, Rehabilitation and Sports Medicine Research Institute of Zhejiang Province, Zhejiang Provincial People’s Hospital (Affiliated People’s Hospital, Hangzhou Medical College), Hangzhou, China; ^2^Department of Rheumatology, Sir Run Run Shaw Hospital, Zhejiang University School of Medicine, Hangzhou, China; ^3^Zhejiang University-University of Edinburgh Institute, Zhejiang University, Hangzhou, China

**Keywords:** osteoarthritis, *FTO*, polymorphism, meta-analysis, systematic review

## Abstract

**Objective:**

The association of fat mass and obesity-related (*FTO*) gene with osteoarthritis (OA) risk has been investigated in multiple genome-wide association studies but showed inconsistent results. Our study aimed to assess *FTO* expression in different OA sequencing datasets and to meta-analyze whether *FTO* polymorphism was associated with the risk of osteoarthritis.

**Method:**

Gene expression profiles were obtained from ArrayExpress, Gene Expression Omnibus (GEO), and BioProject databases. Three electronic databases including PubMed and EMBASE were systematically retrieved to identify articles exploring the association between *FTO* polymorphisms and OA risk published before September 2022. Summary odds ratios (ORs) and corresponding 95% confidence intervals (95% CIs) were calculated to perform the result. Stata software was utilized to conduct analyses on predetermined ethnicity and gender subgroups and sensitivity.

**Results:**

*FTO* gene was differentially expressed in the datasets from the UK. This systematic review and meta-analysis encompasses eight studies that revealed a significant association between *FTO* polymorphisms and OA risk [OR 1.07, 95% CI (1.03, 1.11), *P* < 0.001] in the overall population. In subgroup analysis, a marked association was observed in European Caucasian [OR 1.08, 95% CI (1.04–1.12), *P* < 0.001] and North American Caucasian with the Asian subgroups [OR 0.98, 95% CI (0.83–1. 6), *P* = 0.83] as an exception. Among the studies, four of them demonstrated attenuation in their OA risk after body mass index (BMI) adjustment in Caucasian populations.

**Conclusion:**

*FTO* significant differential expression was associated with the increased risk of OA in Caucasian populations. Nevertheless, the causality between *FTO* polymorphisms and OA risk remains largely elusive. Hence, further studies with larger sample size are necessary to validate whether *FTO* gene polymorphism contributes to OA susceptibility.

## Introduction

Osteoarthritis (OA) is the most prevailing form of whole joint degenerative disease characterized by the degeneration of articular cartilage, bone remodeling, synovial inflammation, osteophyte formation, subchondral sclerosis, infrapatellar fat pad, and meniscus injuries, etc. (1). Its prevalence does not cease to escalate due to population aging, prolonged life expectancy and obesity, making the disease a major healthcare problem and socioeconomic burden affecting millions of people worldwide (1). OA is a multifactorial disease as its pathogenesis is an amalgamative effect of environmental factors such as traumatic joint injury and chronic mechanical overloading alongside genetic risk factors such as aging, gender, genetic predisposition, obesity, and inflammation (2). Previous studies frequently associate obesity with an augmented risk of OA, but how it contributes to the onset and progression of OA has not been well-established (3). On the other hand, it has been demonstrated the presence of OA in non-weight-bearing joints of obese subjects and obesity determines a low-grade inflammatory systemic inflammatory status. Thus, it is suggested that other factors other than mechanical loading contribute to the disease (4, 5).

The fat mass and obesity-associated (*FTO*) gene encodes a 2-oxoglutarate-dependent nucleic acid demethylase which is the first well-established obesity-susceptibility gene (6). Recently, several genome-wide association studies (GWAS) explored the relationship between *FTO* gene variation and OA risk (7–9). However, these studies presented incongruent and inconclusive results attributed to the clinical heterogeneity of patients and various single nucleotide polymorphisms (SNP), different ethnic populations, and small sample sizes. In addition, the microarray and RNA-sequencing data provide us the possibility to investigate whether *FTO* is a candidate gene for OA susceptibility. To precisely elucidate the role of the *FTO* gene in the development of OA, we firstly detected the *FTO* expression between OA and normally followed by a comprehensive meta-analysis to determine the association between *FTO* polymorphisms and OA risk.

## Method

### Search strategy

Microarray and RNA-sequencing data from cartilage samples in OA patients were obtained from ArrayExpress, Gene Expression Omnibus (GEO), and BioProject databases using the search terms “osteoarthritis,” and “cartilage.” We conducted literature searches of databases which include PubMed, EMBASE to retrieve relevant articles that underlined the associations between *FTO* polymorphisms and OA up to September 1, 2022 with “FTO” AND (“OA” OR “osteoarthritis” OR “arthrosis”) as keywords. The search strategy in detail that we performed is illustrated in Supplementary Tables 1, 2. Additionally, the references of related studies were also screened to identify potentially relevant studies. This systematic review and meta-analysis was conducted by adhering to the Preferred Reporting Items for Systematic Reviews and Meta-analyses (PRISMA) reporting guideline (10).

### Inclusion and exclusion criteria

Two investigators assessed the retrieved studies independently according to the pre-specified inclusion criteria as follows: studies that (1) case-control or cohort design; (2) evaluated the association between *FTO* gene polymorphism and knee or hip OA, no limitation in single-nucleotide polymorphisms (SNPs) sites; (3) contained genotype data for the calculation of odds ratios (ORs) and 95% confidence intervals (CIs); (4) were written in English. If several articles reported findings for repeated study populations, we only selected the most recent study or the one with the largest sample size. Any disagreements will be solved by discussion to decide for inclusion or exclusion of the study for the meta-analysis.

### Data extraction and quality assessment

Two investigators extracted the following information from each eligible study independently: first author, year of publication, country, ethnic origin of the study population, names of SNPs, type of OA and sample size, age, female proportion of cases and controls.

Two investigators analyzed the methodological quality of each study by applying the Newcastle–Ottawa Scale (NOS), in terms of the selection of study participants, comparability of outcome groups and outcome measures.

Any disagreements will be resolved by discussion until consensus is reached.

### Statistical analysis

Microarray datasets were obtained using the “GEOquery” R package (11), and after probe id conversion, the “edgR” R package was used to normalize the data with the CPM (computes counts per million) function (12). RNA sequencing datasets were normalized by applying the variance stabilizing transformation (VST) function from the “DESeq2” R package (13). Mann–Whitney *U* test was utilized to compare the *FTO* expression between the OA group and controls. These computational and statistical analyses were performed using the R software.^1^

The odd ratios (ORs) and 95% confidence intervals (CIs) were estimated by the random effects model (DerSimonian and Laird methods) to evaluate the strength of correlation between *FTO* gene polymorphism and OA risk. Stratification analyses were carried out by ethnicity and gender. *P* < 0.05 was considered statistically significant. Sensitivity analysis was performed by repeating analysis after omitting one study each time to estimate the impact on the overall effects. Heterogeneity was assessed by Q statistic with *P*-value and *I*^2^ statistic (14). Potential publication bias will be examined by Egger’s test if more than 10 studies were included (15). These data analyses were performed in Stata 16.0 (Stata Corp, College Station, TX, USA).

## Results

### Fat mass and obesity-related expression between osteoarthritis and control

**FIGURE 1:**
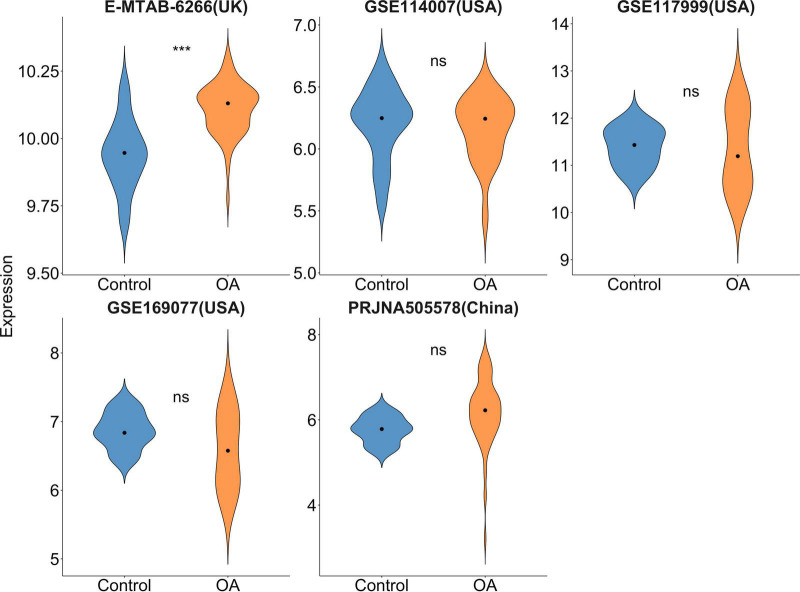
Violin plot of *FTO* gene expression in microarray and RNA-sequencing data. Dots mean median. ***P < 0.001.

A total of 208 records were derived after incipient search. GSE169077 (USA), GSE117999 (USA), GSE11400 (USA), E-MTAB-6266 (UK), and PRJNA505578 (China) datasets were included. Our results (Figure 1) revealed that *FTO* demonstrated a significantly increased differential expression (*P* < 0.001) in the UK OA population but not for the USA or Chinese population (*P* > 0.05).

### Characteristics of the included studies for meta-analysis

**FIGURE 2:**
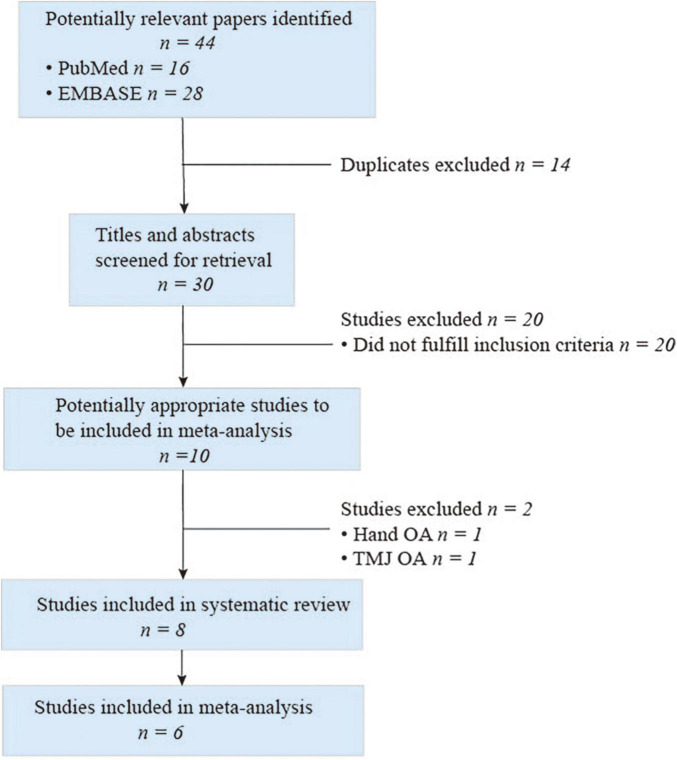
Selection for eligible citations included in this systematic review and meta-analysis.

Selection for qualified studies was presented in Figure 2. Our initial computerized literature search identified a total of 44 citations. Among these results, 14 records were duplication, and 20 records did not meet our inclusion criteria following a thorough review of the titles and abstracts. Ten citations were retrieved for further full-text review; two out of the 10 studies investigated the association of *FTO* polymorphism with hand or temporomandibular joint (TMJ) OA, respectively. Eventually, we identified eight eligible citations for systematic review (7–9, 16–20) and six studies for meta-analysis (7–9, 16–18). The characteristics and quality of these included studies are summarized in Table 1. These available cohort studies were conducted in three countries (number of studies): the UK (2), Finland (1); and China (3) for meta-analysis, and the other two studies synthesized rs8044769 SNP and OA risk in different independent study cohorts (19, 20). Four *FTO* polymorphisms rs8044769 (7, 9, 16, 19, 20), rs12149832 (8), rs9939609 (18), rs1558902 (17) were investigated in this meta-analysis and systematic review. These results of quality assessment were not performed as one studies was from abstract (18) and two studies from several cohorts (19, 20).

**TABLE 1:** Main characteristics of included studies.

References	Country	Ethnicity	SNP	OA status	Sample size	Case			Sample size	Control			NOS
									
						Age	BMI	Female		Age	BMI	Female	
Zeggini et al. (7)	UK	Caucasian	rs8044769	Hip, Knee	7,410	/	/	60.4%	11,009	/	/	50.1%	8
Elliott et al. (8)	UK	Caucasian	rs12149832	Hip, Knee, K/L grade ≥ 2	/	/	/	/	/	/	/	/	8
Welling et al. (18)	Finland	Caucasian	rs9939609	Knee	402	/	/	/	5,348	/	/	/	/
Panoutsopoulou et al. (19)	UK and Australia	Caucasian	rs8044769	Hip, Knee, K/L grade ≥ 2	9,764	/	/	/	5,362	/	/	/	/
Wang et al. (9)	China	Asian	rs8044769	Knee, K/L grade ≥ 2	196	62.19 ± 8.76	/	76%	442	57.17 ± 9.19	/	69%	8
Yau et al. (20)	USA	Caucasian	rs8044769	Hip, Knee, K/L grade ≥ 2	3,898	/	/	/	3,168	/	/	/	/
Dai et al. (16)	China	Asian	rs8044769	Knee	890	62.51 ± 11.43	25.76 ± 3.69	75%	844	54.07 ± 11.60	24.91 ± 3.04	20%	8
Li et al. (17)	China	Asian	rs1558902	Knee K/L grade ≥ 1	532	58.1 ± 7.2	23.9 ± 4.1	60%	927	57.5 ± 8.9	23.4 ± 6.3	63%	7

BMI, body mass index (kg/m^2^); K/L grade, Kellgren–lawrence (K/l) grading system.

### Meta-analysis of fat mass and obesity-related gene polymorphism and osteoarthritis risk in all population

**FIGURE 3:**
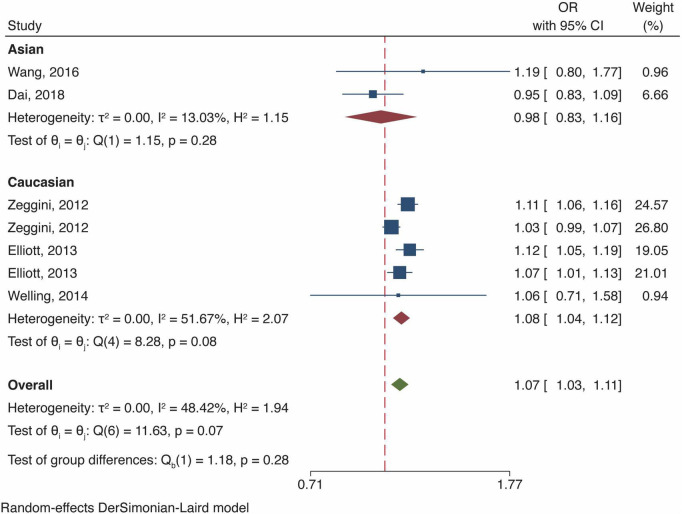
Meta-analysis of *FTO* gene polymorphism and OA risk in all population.

In the general analysis, we found that *FTO* gene polymorphism increased OA risk [OR and 95% CI, 1.07 (1.03, 1.11), *P* < 0.001, Figure 3] with accept heterogeneity (*I*^2^ = 48.42%). Stratified analysis of ethnicity showed that the risk of OA was considerably elevated by *FTO* polymorphism thein European Caucasian (OR 1.08 [95% CI 1.04–1.12], *P* < 0.001, *I*^2^ = 51.67%, Figure 3) but did not reveal a statistically significant rise in Asian (OR 0.98 [95% CI 0.83–1.16] *P* = 0.83, Figure 3) with low heterogeneity (*I*^2^ = 13.03%). Meanwhile, Yau et al. documented that *FTO* polymorphism (rs8044769) increased OA risk in North American Caucasian (OR 1.10 [95% CI 1.03–1.19], *P* = 0.00613) (20). Four studies investigated the effect of body mass index (BMI) covariate on the OA outcome, consistent herewith, we observed an attenuation of the OA risk after BMI adjustment in the Caucasian population (7, 8, 18, 19). Nonetheless, we did not discover any solid association between *FTO* polymorphism (rs8044769) and higher BMI in the Chinese population (9, 16).

### Meta-analysis of fat mass and obesity-related gene polymorphism and osteoarthritis risk in female population

**FIGURE 4:**
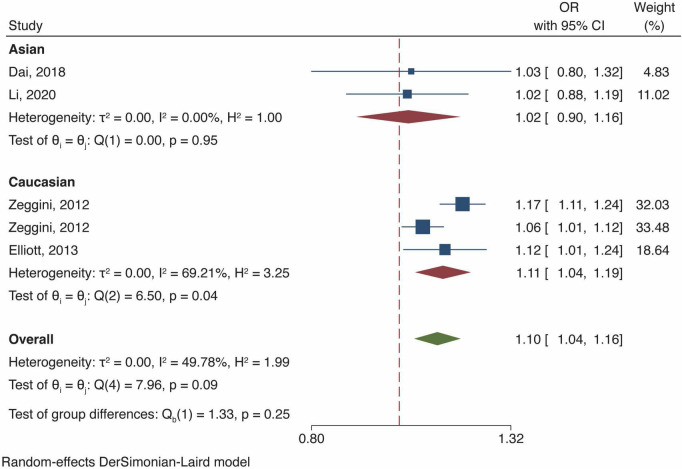
Meta-analysis of *FTO* gene polymorphism and OA risk in female population.

Four studies investigated the association of *FTO* polymorphism and OA risk in female population, all of which reported results that ascribed the increase in OA risk to *FTO* gene polymorphism [OR and 95% CI, 1.10 (1.04, 1.16), *P* < 0.01, Figure 4]. The ethnic-stratified analysis demonstrated that FTO polymorphisms significantly augmented the OA risk in European Caucasian, with Asian as the exception, which was consistent with the overall population (Figure 4).

### Sensitivity analysis and publication bias

The leave-one-out analysis in all populations revealed that no single study changed pooled ORs (Supplementary Table 3), indicating the statistical robustness of our results. Since only six studies were included, we would not carry out the publication bias.

## Discussion

To our best knowledge, this is the first meta-analysis that incorporated *FTO* gene expression data to evaluate the association between *FTO* gene polymorphism and OA susceptibility. Our results revealed that *FTO* polymorphism-induced OA risk increase was significant in European Caucasian but not in Asian populations, which is consistent with the results of *FTO* exhibiting significant differential expressions in the UK population but not in Chinese population. The association strength of *FTO* polymorphism and risk of OA attenuated after BMI adjustment in Caucasian population.

Fat mass and obesity-related polymorphism manifests differences between Asian and Caucasian populations. This is possibly owing to the fact that ethnic heritage and geographic localization are major influential factors contributing to genetic polymorphisms, which could render a difference in allele frequency (21). However, this result may also be affected by other confounding factors. The sample size of the Chinese population might not be statistically large enough to reach a convincing conclusion. On the other hand, selection bias in patient enrolment and differences in OA-occurring joint sites could potentially undermine the robustness of the findings. Panoutsopoulou et al. examined the strength of association of rs8044769 with knee or hip OA (adjusted for gender) and detected a distinct association of the FTO variant with knee OA (OR 1.08 [95% CI 1.02–1.14], *P* = 0.009) rather than hip OA (OR 1.04 [95% CI 0.98–1.11], *P* = 0.17) in Caucasian populations. For non-weight-bearing joints such as hand and TMJ OA, *FTO* polymorphism also increased the OA risk (22, 23). These findings require further validation in the future with larger-scale observational studies.

Fat mass and obesity-related is an obesity susceptibility gene, but its mechanism on OA is still controversial. Our results showed that the association signal was fully attenuated after BMI adjustment, insinuating the possibility that the *FTO* gene exerts its effect on OA through obesity in the Caucasian population. However, in the Asian population, the relationship between *FTO* gene polymorphism and obesity remained ambiguous. Our results illustrated that there is no solid association between *FTO* polymorphism and higher BMI in the Chinese population, which is contrary to the result of Chang et al. (24). Consistent herewith, the Japanese studies also failed to demonstrate the association of *FTO* polymorphism with obesity or BMI in their population (25–27). Since the Asian population, generally, is lighter than the UK and even more the USA one; it is possible the presence of a bias due to this difference in BMI of the different populations. On the other hand, these may be due to the sample selection bias for study subjects or genetic variants in *FTO*, and further studies are necessary to contemplate the association of *FTO* with BMI and the risk of OA in the Asian population. Meanwhile, more research needs to fill the gaps in the association between *FTO* polymorphism and OA risk in the African population. In addition, FTO plays an important role in N^6^-methyladenosine (m^6^A) modification. m^6^A modification affects the stability and function of RNAs through the “writers,” “erasers,” and “readers” proteins (28). Several studies reinforced this concept by proving that METTL3 which is the “writer” of m^6^A, could limit OA progression by inhibiting m^6^A expression (29). Herein, FTO, as the “eraser” of m^6^A, has the ability to remove the m^6^A modification. As such, FTO should therefore be fully investigated for its role in the onset and progression of OA.

Nevertheless, there are some limitations in this meta-analysis. First, due to limited data, we were unable to conduct further stratification analyses of other potential risk factors, such as age, type of SNPs, BMI, and OA site. On top of that, we could not perform a meta-analysis using a dominant model or recessive model. Second, some studies shared the study subjects of control group, which may lead to bias in the final results albeit the fact that a sensitivity analysis was conducted. Third, our results were predominantly based on unadjusted estimates for confounding factors, which might have affected the final results. Fourth, the exclusive inclusion of articles written in English but no other languages in this study might have introduced selection bias.

In conclusion, this meta-analysis confirms that *FTO* gene polymorphism increased OA risk. Stratification analysis of ethnicity revealed that the augmented risk of OA due to *FTO* polymorphism may exert its effect through obesity in the Caucasian population. Further studies with larger sample size are necessary to validate whether *FTO* gene polymorphisms contribute to OA susceptibility with an emphasis on studying Asian and African populations.

## Data availability statement

The raw data supporting the conclusions of this article will be made available by the authors, without undue reservation.

## Author contributions

KZ and PS conceived and designed the meta-analysis. KZ, LN, and PS performed the literature search and analyzed the data. KZ wrote the manuscript and XY revised it. GC polished the language. All authors contributed to the article and approved the submitted version.

## Footnotes

1
https://www.r-project.org/
