# Supplementary material for: Association between fat mass and obesity-related variant and osteoarthritis risk: Integrated meta-analysis with bioinformatics
Source: Front Med (Lausanne). 2022 Sep 23;9:1024750. doi: 10.3389/fmed.2022.1024750 (PMC9537627; doi:10.3389/fmed.2022.1024750)
Supplement: Supplementary file 1 [file Data_Sheet_1.docx]

**Supplementary Data**

**Table S1. Search strategy for sequencing data**

***Gene Expression Omnibus (GEO) database:***

("osteoarthritis"[MeSH Terms] OR osteoarthritis[All Fields]) AND ("cartilage"[MeSH Terms] OR cartilage[All Fields]) AND ("gse"[Filter] AND "Homo sapiens"[Organism] AND ("Expression profiling by array"[Filter] OR "Expression profiling by high throughput sequencing"[Filter]))

Results:63

Included accession: GSE169077- GSE117999- GSE114007

***ArrayExpress database:***

Osteoarthritis- Filtered by organism Homo sapiens

Results:76

Included accession: E-MTAB-6266

**Bioporject database:**

osteoarthritis [All Fields] AND cartilage [All Fields] AND "org human"[Filter]

Results：69

Included accession: PRJNA505578

**Table S2. Search strategy for meta-analysis**

| **Database** | **#** | **Search strategy** | **Results** |
| --- | --- | --- | --- |
| PubMed | 1 | "FTO protein- human" [Supplementary Concept] | 1,256 |
|  | 2 | "Alpha-Ketoglutarate-Dependent Dioxygenase FTO"[MeSH] | 1,438 |
|  | 3 | "Alpha Ketoglutarate Dependent Dioxygenase"[Title/Abstract] OR "Fat Mass and Obesity Associated"[Title/Abstract] OR "AlkB Homolog 9"[Title/Abstract] | 1,114 |
|  | 4 | FTO[Title/Abstract] OR KIAA1752[Title/Abstract] OR ALKBH9[Title/Abstract] OR MGC5149[Title/Abstract] OR rs8044769[Title/Abstract] OR rs12149832[Title/Abstract] OR rs9939609[Title/Abstract] OR rs1558902[Title/Abstract] | 3,851 |
|  | 5 | #1 OR #2 OR #3 OR #4 | 4,064 |
|  | 6 | “Osteoarthritis” [MeSH] | 73,664 |
|  | 7 | osteoarthr*[Title/Abstract] OR arthrosis[Title/Abstract] or OA [Title/Abstract] | 108,412 |
|  | 8 | degenerative [Title/Abstract] AND (arthritis [Title/Abstract] or “joint disease” [Title/Abstract]) | 6,315 |
|  | 9 | #6 OR #7 OR #8 | 130,793 |
|  | 10 | #5 AND #9 | 16 |
| Embase | 1 | 'alpha ketoglutarate dependent dioxygenase FTO'/exp | 999 |
|  | 2 | 'alpha ketoglutarate dependent dioxygenase':ab-ti OR 'fat mass and obesity associated':ab-ti OR 'alkb homolog 9':ab-ti | 1,219 |
|  | 3 | fto:ab-ti OR kiaa1752:ab-ti OR alkbh9:ab-ti OR mgc5149:ab-ti OR rs8044769:ab-ti OR rs12149832:ab-ti OR rs9939609:ab-ti OR rs1558902:ab-ti | 4,348 |
|  | 4 | #1 OR #2 OR #3 | 4,592 |
|  | 5 | 'osteoarthritis'/exp | 153,968 |
|  | 6 | osteoarthr*:ab-ti OR arthrosis:ab-ti OR oa:ab-ti | 149,349 |
|  | 7 | degenerative:ab-ti AND (arthritis:ab-ti OR 'joint disease':ab-ti) | 8,395 |
|  | 8 | #5 OR #6 OR #7 | 205,382 |
|  | 9 | #4 AND #8 | 28 |

Search Date: September 1^st^, 2022

**Table S3. Results of sensitivity analyses with exclusion of the listed studies**

| **Studies** | **Ethnicity** | **OR (95 CI)** | ***P* value** | **I^2^(%)** |
| --- | --- | --- | --- | --- |
| None | Overall | 1.07 (1.03 - 1.11) | < 0.001 | 48.42 |
|  | Caucasian | 1.08 (1.04 - 1.12) | < 0.001 | 51.67 |
|  | Asian | 0.98 (0.83 -1.16) | 0.83 | 13.03 |
| Zeggini- 2012a | Overall | 1.06 (1.01 - 1.10) | 0.01 | 37.58 |
|  | Caucasian | 1.07 (1.02 - 1.11) | 0.0033 | 42.59 |
|  | Asian | 0.98 (0.83 - 1.16) | 0.83 | 13.03 |
| Zeggini- 2012b | Overall | 1.09 (1.05 - 1.13) | < 0.001 | 16.46 |
|  | Caucasian | 1.10 (1.07 - 1.13) | < 0.001 | 0 |
|  | Asian | 0.98 (0.83 - 1.16) | 0.83 | 13.03 |
| Elliott- 2013a | Overall | 1.06 (1.02 - 1.11) | 0.0086 | 45.25 |
|  | Caucasian | 1.07 (1.02 - 1.11) | 0.0022 | 50.87 |
|  | Asian | 0.98 (0.83 - 1.16) | 0.83 | 13.03 |
| Elliott- 2013b | Overall | 1.07 (1.02 - 1.13) | 0.01 | 57.02 |
|  | Caucasian | 1.08 (1.03 - 1.14) | 0.003 | 63.70 |
|  | Asian | 0.98 (0.83 - 1.16) | 0.83 | 13.03 |
| Welling- 2014 | Overall | 1.07 (1.03 - 1.12) | 0.0014 | 57.02 |
|  | Caucasian | 1.08 (1.04 - 1.12) | < 0.001 | 63.74 |
|  | Asian | 0.98 (0.83 - 1.16) | 0.83 | 13.03 |
| Wang- 2016 | Overall | 1.07 (1.03 - 1.11) | 0.0014 | 55.95 |
|  | Caucasian | 1.08 (1.04 - 1.12) | < 0.001 | 51.67 |
|  | Asian | 0.95 (0.83 - 1.09) | / | / |
| Dai- 2018 | Overall | 1.08 (1.04 - 1.12) | < 0.001 | 41.46 |
|  | Caucasian | 1.08 (1.04 - 1.12) | < 0.001 | 51.67 |
|  | Asian | 1.19 (0.8 - 1.76) | / | / |

*a- b means there are two included datasets in one study.
